# Low-temperature-induced changes in the transcriptome reveal a major role of *CgSVP* genes in regulating flowering of *Cymbidium goeringii*

**DOI:** 10.1186/s12864-019-5425-7

**Published:** 2019-01-17

**Authors:** Fengxi Yang, Genfa Zhu, Yonglu Wei, Jie Gao, Gang Liang, Lingyuan Peng, Chuqiao Lu, Jianpeng Jin

**Affiliations:** 1 Guangdong Key Laboratory of Ornamental Plant Germplasm Innovation and Utilization, Environmental Horticulture Research Institute, Guangdong Academy of Agricultural Sciences, Guangzhou, 510640 People’s Republic of China; 2 CAS Key Laboratory of Tropical Plant Resources and Sustainable Use, Xishuangbanna Tropical Botanical Garden, Kunming, Yunnan 650223 People’s Republic of China

**Keywords:** *Cymbidium goeringii*, Low-temperature, Flowering, Transcriptome, SVP

## Abstract

**Background:**

*Cymbidium goeringii* is one of the most horticulturally important and popular ornamental plants in the orchid family (Orchidaceae). It blooms in winter during January–March and a period of low temperature is necessary for its normal flowering, otherwise there is flower bud abortion, which seriously affects the economic benefits. However, the molecular mechanism underlying winter-blooming behavior in *C. goeringii* is unclear.

**Results:**

In this research, we firstly study the flowering physiology of *C. goeringii* by cytobiology observations and physiological experiments. Using comparative transcriptome analysis, we identified 582 differentially expressed unigenes responding to cold treatment that were involved in metabolic process, flowering time, hormone signaling, stress response, and cell cycle, implying their potential roles in regulating winter-blooming of *C. goeringii*. Twelve MADS-box genes among them were investigated by full-length cDNA sequence analysis and expression validation, which indicated that three genes within the SHORT VEGETATIVE PHASE (SVP) sub-group had the most significant repressed expression after cold treatment. Further analysis revealed that the SVP genes showed population variation in expression that correlated with cold-regulated flowering and responded to low temperature earlier than the flowering pathway integrators *CgAP1*, *CgSOC1*, and *CgLFY*, suggesting a potential role of *CgSVP* genes in the early stage of low-temperature-induced blooming of *C. goeringii.* Moreover, a yeast two-hybrid experiment confirmed that CgSVP proteins interacted with CgAP1 and CgSOC1, suggesting that they may synergistically control the process of *C. goeringii* flowering in winter.

**Conclusions:**

This study represents the first exploration of flowering physiology of *C. goeringii* and provides gene expression information that could facilitate our understanding of molecular regulation of orchid plant winter-flowering, which could provide new insights and practical guidance for improving their flowering regulation and molecular breeding.

**Electronic supplementary material:**

The online version of this article (10.1186/s12864-019-5425-7) contains supplementary material, which is available to authorized users.

## Background

The family Orchidaceae includes some of the most horticulturally important and popular ornamental plants in the world [1, 2]. Particularly, the genus *Cymbidium*, which includes *C. goeringii*, *C. sinense*, *C. faberi* Rolfe, *C. tortisepalum*, and *C. kanran* Makino, holds a strong position in the traditional flower market in China, Japan, Korea, and Southeast Asia [3, 4]. Most of these orchids bloom in winter during January–March and need 5–10 °C low-temperature conditions to promote flower opening. *Cymbidium goeringii*, which is typically characterized by low-temperature-induced blooming, is considered a Spring Festival flower in most Asian countries [5–7]. As a low-growing perennial herb, *C. goeringii* needs long photoperiods to induce floral spiking initiation from June, and then the initiated floral buds grow very slowly and become semi-dormant in the following months. By winter, a period of low temperatures is needed to end this slow-growth stage and promote inflorescence elongation and flower opening, otherwise this will result in flower bud abortion and seriously affect economic benefits. The process of flower initiation to flowering needs about 6 months, and a low-temperature period is critical for blooming. This greatly restricts the planting area of *C. goeringii*. Unfortunately, a way to genetically manipulate the flowering time of these orchids independently of temperature control is still unavailable. Understanding how flowering time and floral organ development are regulated by ambient temperature will provide practical guidance for molecular breeding and is of great importance to the orchid industry.

Molecular mechanisms underlying flowering regulation have been extensively studied in model annual plants such as long-day plant *Arabidopsis* [8, 9], short-day plant rice [10], and winter cereals wheat [11, 12] and barley [13]. However, in perennials, which have typical growth cycles including the juvenile to adult and vegetative to reproductive transitions, and flower initiation with flowering completion in the next year, understanding of the molecular mechanisms controlling flowering has just started to emerge. Recent studies in perennials, such as apple, peach and poplar, have revealed striking similarities between genes and pathways that regulate flowering time in annuals and growth and dormancy cycles in perennials. As reviewed by Ding and Nilsson (2016) [14], homologs of circadian-clock and photoperiod pathway key components phytochromes (PHY) and CONSTANS (CO) impact on growth cessation; and homologs of flowering integrator gene *FLOWERING LOCUS T* (*FT*), bZIP transcription factor *FD*, and floral meristem identity gene *APETALA1* (*AP1*) have all been shown to control the onset of reproduction.

Some other genes, such as those encoding APETALA2/Ethylene responsive transcription factor EARLY BUD-BREAK 1 (EBB1) and the C-repeat binding factor (CBF/DREB), were also identified to determine the time of bud break in trees [15, 16]. Among the transcription factors, an important class of genes clearly associated with regulation of flowering in annuals and dormancy in perennials are MADS-box genes with similarity to *Arabidopsis SHORT VEGETATIVE PHASE* (*SVP*)*.* For example, in annuals, *Arabidopsis SVP* is involved in both temperature- and photoperiod-dependent flowering pathways, and it forms a complex with *FLOWERING LOCUS C* (*FLC*) and *FLOWERING LOCUS M* (*FLM*), resulting in repression of *FT* and flowering [17–19]. Barley SVP-group member *BM1* is induced by cold treatment and causes a slight delay in heading time [20]. In contrast, the wheat SVP-group gene *TaVRT2* is suppressed by vernalization, suggesting a differential expression profile in adaptation to the environment [21, 22]. In perennials, the peach *SVP* gene family is expanded to six *DAM* genes, and their expression tracks seasonal light and temperature cycles, integrating environmental cues that regulate the transition into and out of endodormancy [23, 24]. *Prunus mume SVP* gene *DAM6* caused early growth cessation and early terminal bud set [25]. *Actinidia chinensis SVP2* delayed budbreak in transgenic *A. deliciosa* and *SVP3* significantly repressed flower development, delayed the time of flower opening, and diminished flower fertility in *A. eriantha* as well [26, 27]. These results demonstrate similar molecular activity and growth inhibitory function among *SVP-like* genes. However, differential roles have been found for individual *SVP* gene family members, including bud dormancy, flowering initiation, and floral development – probably related to the matching of plant growth period in adapting to local environments and seasonal changes.

In order to understand the molecular mechanisms underlying the winter-flowering behavior of *C. goeringii*, based upon data from field observations and physiological experiments, we found at least 40-day of 10 °C night-temperature was required to overcome the slow-growing stage, which could promote flowering rate from less than 20 to 85% and make flowers bloom 30 days in advance. By comparative analysis of the floral bud transcriptome after 40 days of low-temperature treatment, we identified 582 unigenes probably related to *C. goeringii* flowering regulation. The genes were involved in metabolic pathways, flowering time, hormone signaling, stress response, and cell cycle. Among them, 12 MADS-box genes were further investigated by full-length cDNA sequence analysis and expression validation. Particularly, three genes of different sub-clades within the *CgSVP* clade were characterized. Expression of *CgSVP* genes showed population and cultivar variation that correlated well with cold-regulated flowering. Results from yeast two-hybrid screening further indicated that the CgSVP proteins interacted with CgAP1 and CgSOC1, implying their potential roles in the control of *C. goeringii* flowering process. These results represent the first exploration of flowering physiology of *C. goeringii* and reveal the crucial role of *CgSVP* genes regulating its winter-flowering behavior, which shed light on the molecular regulation of winter-flowering of the orchid plant, and provide new insights and practical guidance for improving their flowering regulation and molecular breeding.

## Materials and methods

### Plant materials and growth conditions

Wild-type plants of *C. goeringii* ‘Songmei’ (the most widely known commercial cultivar in China) used in this study were artificially cultivated and collected from the cultivation base of the Environmental Horticulture Research Institute, Guangdong Academy of Agricultural Sciences, China. Cold treatment of day/night with temperatures of 25/10 °C and photoperiod of 16/8 h was carried out from September to November, 2015. The plants were classified into four groups with a prolonged cold treatment of a further 20, 30, 40, and 50 -day and then returned to normal conditions of 25/20 °C.

### Library construction and Illumina sequencing

The primary aim of this study was to identify the genes responsible for low-temperature-induced flowering. The cDNA libraries were prepared from RNAs isolated from floral buds before and after 40-day cold treatment. The mRNAs were purified from total RNA using the Oligotex mRNA Midi Kit (QIAGEN, Germany) and quantified using a Nano-Drop 2000 spectrophotometer (Thermo Scientific, USA) to generate the cDNA library according to the Illumina manufacturer’s instructions as in our previous work [28]. Briefly, mRNAs were isolated from Total RNA and fragmented to approximately 200 bp. Subsequently, the collected mRNAs were subjected to first strand and second strand cDNA synthesis following by adaptor ligation and enrichment with a low-cycle according to instructions of TruSeq® RNA HT Sample Prep Kit (Illumina, USA). The purified library products were evaluated using the Agilent 2200 TapeStation and Qubit®2.0(Life Technologies,USA) and then diluted to 10 pM for cluster generation in situ on the HiSeq2500 pair-end flow cell followed by sequencing (2 × 100 bp). An average of more than 60 million reads was generated for each sample.

### DEG analysis

The gene expression level was calculated by the RPKM value: RPKM = [total exon reads / mapped reads (millions)] × exon length (kb). The significance of differences in gene expression between the wild-type and mutant were determined using edgeR. The false discovery rate (FDR) was applied to identify the threshold of the *P*-value in multiple tests; with FDR < 0.05 and |log2 ratio| > 1 (two-fold change) as the threshold for a significant difference in gene expression.

The differentially expressed genes (DEGs) were annotated using gene ontology (GO) and Kyoto Encyclopedia of Genes and Genomes (KEGG) enrichment analyses according to a method similar to that described by Wang et al. (2014) [29], which firstly mapped all DEGs to GO terms (or KEGG pathways) in the databases (http://www.geneontology.org/ or http://www.genome.ad.jp/) and calculated gene numbers for every term (or pathway) [30, 31]. This was followed by a hypergeometric test to find the significantly enriched terms in DEGs compared with the genome background. We used the corrected *P*-value ≤0.05 or Q-value ≤0.05 as a threshold for the significantly enriched GO terms or KEGG pathways, respectively, in DEGs.

### Real-time quantitative RT-PCR (qRT-PCR) analysis

For the qRT-PCR of target genes, total RNA extracted from different tissue types was reverse-transcribed using a oligo(dT) primed cDNA synthesis protocol (Fermentas). The resulting cDNA was subjected to relative quantitative PCR using Bio-Rad CFX-96 RealTime PCR System (Bio-Rad, USA) in a final volume of 20 μl containing 2 μl of cDNA and 10 μl of SYBR premix Ex-taq™ (Takara, Japan). *Ubiquitin* and *Actin* were used as internal controls for normalization to make a comparison of gene expression level between the accessions. For each reported result at least three independent biological samples were subjected to a minimum of three technical replicates. The primers designed with Primer 5.0 software are listed in Additional file 1: Table S1. Genbank accession numbers of the MADS-box genes examined are MF474250, MF474239, MF474243, MF462094, MF462081, MF462082, MF462083, MF474256, MF474244, MF474248, and MF462093.

### Yeast two-hybrid assay

For the yeast two-hybrid assay, full-length CDs of *CgSVP1* and *CgSVP2* were cloned into pGBKT7, and full-length *CgSVP1*, *CgSVP2*, *CgAP1*, and *CgSOC1* were cloned into pGADT7, respectively. Growth was determined as described in the Yeast Two-Hybrid System User Manual (Clontech). Experiments were repeated three times.

### Transient gene expression assays

Infiltrations were performed according to a method modified from that previously described by Wroblewski et al. (2010) [32]. *Agrobacterium* was resuspended with infiltration solution (pH =5.6) which contains 10 mm 2-(N-morpholino) ethanesulphonic acid (MES), 10 mm magnesium chloride (Mgcl2), and 100 μm Acetosyringone (AS). Bacterial densities were adjusted to OD600 = 0.4–0.5 prior to infiltration. 500 μl of the *Agrobacterium* suspension contained in the syringe was then ejected into the floral bud where it was rapidly absorbed and repeated the experiment 7 days later. GFP activity was visualized using a FluoroImager (Leica Microsystems, DFC7000T).

### Scanning electron microscopy

Lateral buds were collected weekly from 20 June to 1 September, and periodically thereafter until the next spring. Buds were partially dissected by removing outer bud scales, and the dissected apices were fixed in a solution of 3% glutaraldehyde and 2% formaldehyde for 24 h. Then samples were dehydrated in acetone, critical-point dried in liquid CO_2_, and mounted on stubs and sputter coated with 25 nm gold. Finally the samples were examined by scanning electron microscope JSM-6360LV (JEOL).

## Results

### Flowering physiology of *C. goeringii*

**Fig. 1:**
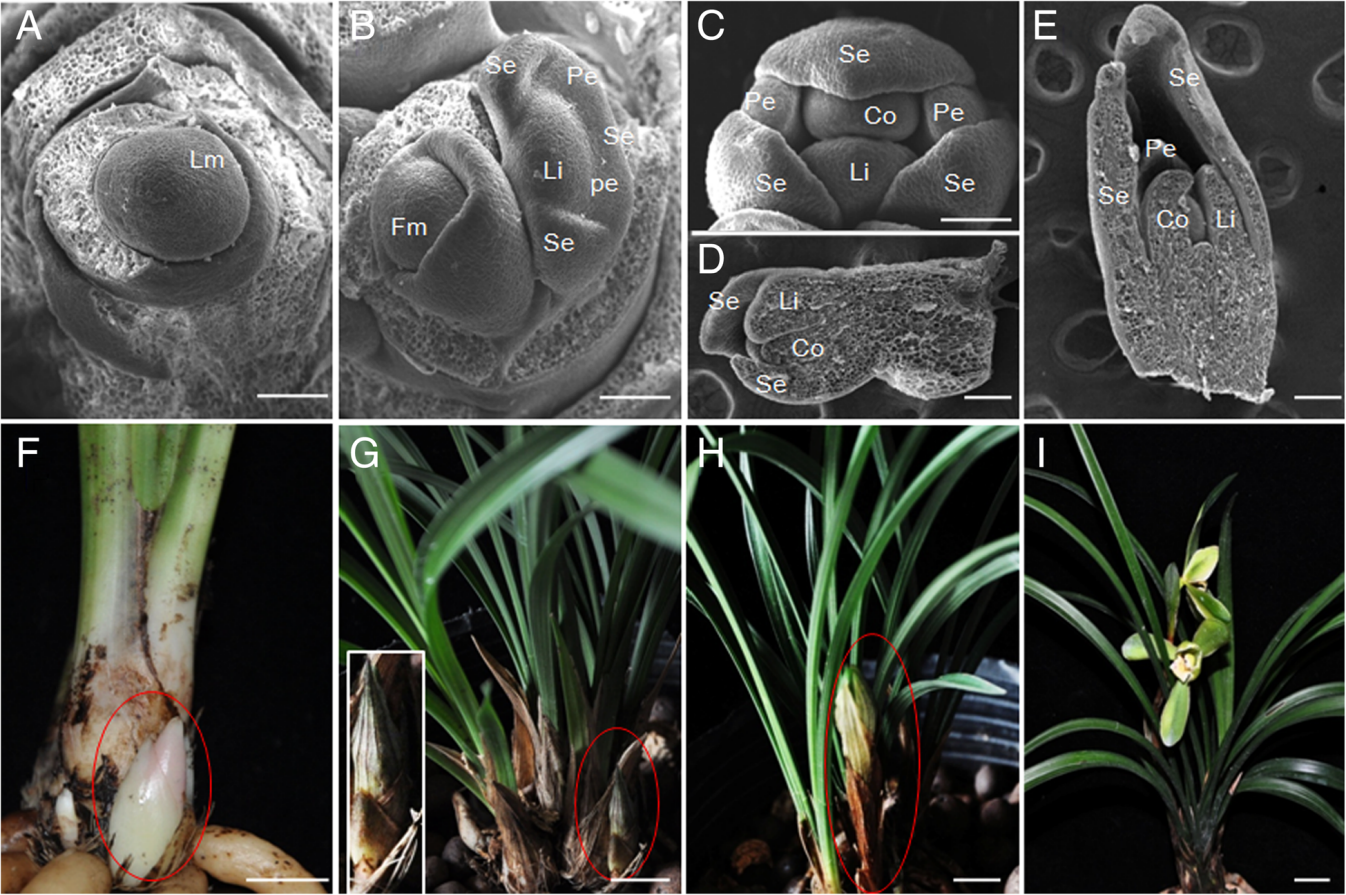
Floral development of *Cymbidium goeringii.* **a**–**e** Scanning electron micrograph (SEM) of early floral developmental stages of wild-type *C. goeringii.* Bar = 100 mm. Im, inflorescence meristem; Fm, floral meristem; Se, sepal primordium; Pe, petal primordium; Li, lip primordium; Co, column primordium. (**f**-**h**) developing floral bud, Bar = 1 cm. **f** potential floral bud initiated in the lateral buds of developing shoots. **g** semi-endodormant floral bud. **h** developing bud after cold-treatment in winter. **i** blooming flower

Meristems with a potential to differentiate flowers are initiated in the lateral buds of developing shoots of *C. goeringii* from July. The initiated floral buds grow very slowly and become semi-dormant in the following months. We recorded the morphogenesis, growth rate, and surface structure of floral organs in detail using scanning electron microscopy. Flower development is divided into five stages (Fig. 1). Stage 1 begins with the initiation of a floral buttress on the flank of the shoot and forms a flower primordium (Fig. 1a and f). Stage 2 commences very soon after the flower primordium divides into sepal primordia and petal stamen (Fig. 1b). Gynostemium primordia appear next (stage 3, Fig. 1c and d) and petal primordia grow rapidly (stage 4, Fig. 1e); then the gynostemium primordia enlarge very slowly for a long period of 5 months which we named as semi-endodormancy (Fig. 1g and h) and the final stage (stage 5) ends when the flower opens (Fig. 1i).

**Fig. 2:**
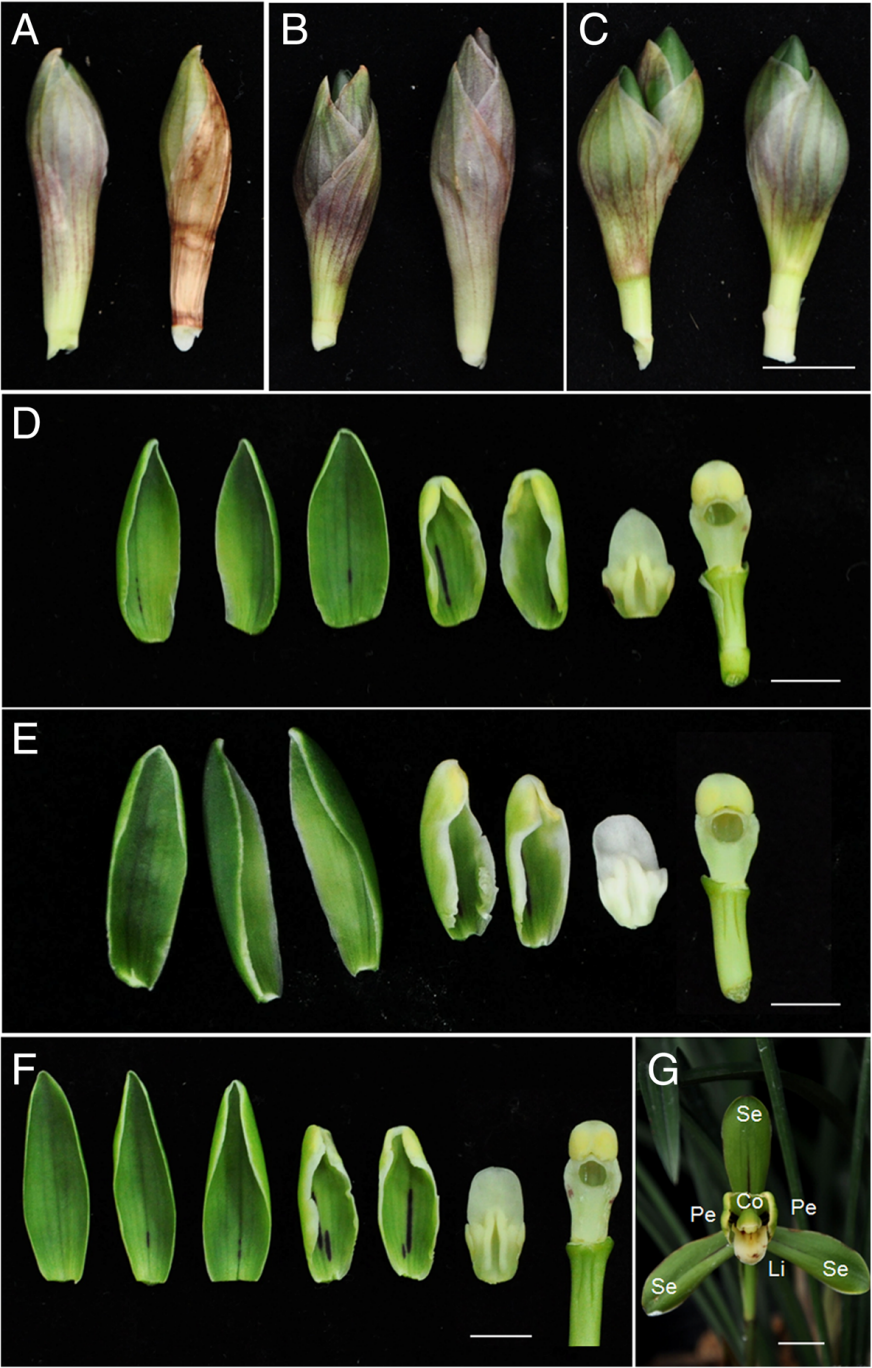
Cold pre-treatment and floral development stage of *Cymbidium goeringii.* **a**–**c** 2 representative floral buds of the plants treated with a prolonged cold condition of 0, 20, and 40 days, respectively, and then returned to normal condition. **d**–**f** Developing floral organs the plants treated with a prolonged cold condition of 0, 20, and 40 days, respectively, and then returned to normal condition. **g** blooming flower. Se, sepal; Pe, petal; Li, lip; Co, column, Bar = 1 cm

Under natural growth conditions it took 6–7 months to progress through the five stages from initiation to flower opening. Cold conditions were needed to avoid bud abortion (Fig. 2a, b and c) and promote flower development (Fig. 2d, e, f and g) in the period of semi-endodormancy. As shown in Table 1, when exposed to 6–8 weeks of prolonged 10 °C night-temperature treatment, flower buds on plants showed accelerated shoot growth and more than 85% flowering after their return to growth-promoting conditions, compared with 18.4% of those without cold treatment. These results indicated that prolonged cold treatment of semi-endodormant floral buds resulted in a dual response that ended the slow-growing stage, and at least 40 days was needed to fulfill the cold requirement.

**Table 1:** Flowering physiology of *Cymbidium goeringii*

Treat-ment (day)	Flowering rate(%)	Early blooming (day)	pedicel length (cm)	Pedicel diameter (mm)	Sepal width (mm)	Sepal length (mm)	Petal width (mm)	Petal length (mm)
0	18.4	71 ± 5.9	11.40	3.17	1.23	2.78	1.31	3.68
20	30.8	64 ± 4.3	10.96	3.03	1.12	2.82	1.26	3.74
30	54.7	50 ± 5.2	10.13	3.34	1.10	2.60	1.23	3.59
40	84.5	40 ± 4.4	10.40	3.09	1.13	2.73	1.25	3.64
50	85.8	42 ± 3.9	9.53	3.16	1.08	2.58	1.28	3.72

Early blooming day represents the number of days after the cold treatment to fully open flowers

### Transcriptome sequencing and DEGs

To generate the inventory of gene expression during cold-induced flowering, we collected floral buds before and after cold treatment for comparative analyses by constructing poly-A RNA-seq libraries on an Illumina platform. This generated total reads of 59,201,226 and 63,343,254 in floral buds before and after cold treatment, respectively, with corresponding clean reads of 55,771,784 (94.21%) and 59,251,576 (93.54%) (Table 2). Considering that a reference genome sequence of *C. goeringii* is lacking, we firstly de novo assembled the total 1115.02 million clean reads and obtained a dataset of 67,854 unigenes with a mean length of 903.2 bp, which is less than our previous de novo dataset of *C. goeringii* flowers that contains 98,446 unigenes with a mean length of 989 bp [5]. To maximize the exploration of transcripts expressed in the floral bud before and after cold treatment, we therefore mapped all sequencing reads to *C. goeringii* floral transcriptome comprising 98,446 unigenes reported in our previous work. Total mapped reads were 44,370,452 (79.56%) and 48,590,106 (82.01%), respectively. This suggests that the majority of transcriptionally active genes were captured in our initial transcriptome assembly (NCBI Genbank accession number SRP091422).

**Table 2:** Summary of sequencing data

	Ck	Cold
Total reads	59,201,226	63,343,254
Clean reads	55,771,784 (94.21%)	59,251,576 (93.54%)
clean_data_base	5,539,214,099 (93.57%)	5,884,364,132 (92.90%)
Total mapped	44,370,452 (79.56%)	48,590,106 (82.01%)
Multiple mapped	11,474,064 (20.57%)	13,179,113 (22.24%)
Uniquely mapped	32,896,388 (58.98%)	35,410,993 (59.76%)
Read1 mapped	22,194,071 (39.79%)	24,307,230 (41.02%)
Read2 mapped	22,176,381 (39.76%)	24,282,876 (40.98%)
Reads map to ‘+’	22,124,348 (39.67%)	24,215,890 (40.87%)
Reads map to ‘-’	22,246,104 (39.89%)	24,374,216 (41.14%)
Reads mapped in proper pairs	20,253,005 (36.31%)	22,432,511 (37.86%)

For the estimation of overall transcriptional activity in floral buds before and after cold treatment, we determined reads per kilobase per million of mapped reads (RPKM) for each gene and calculated the number of DEGs (RPKM ≥1) based on RPKM value. Overall, 582 genes showed altered expression after cold treatment, with 381 up-regulated and 201 down-regulated (Additional file 2: Table S2), suggesting they had a potential role in cold response and flower development.

### Annotation statistics and functional enrichment

**Fig. 3:**
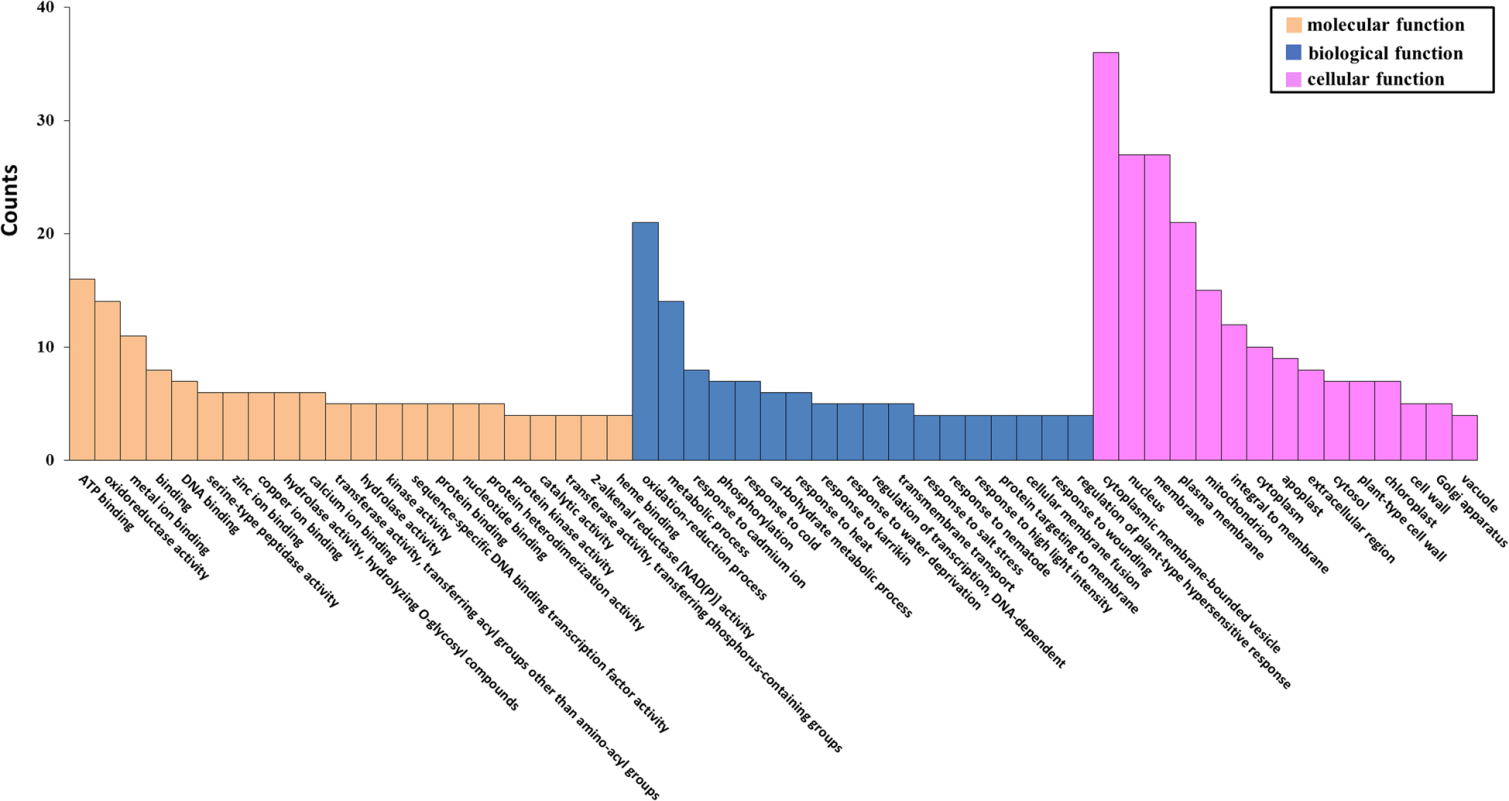
GO classification of unigenes differentially expressed after cold treatment

To reveal the major functional categories represented in these genes, we performed GO enrichment analysis and assigned 55 GO terms (Fig. 3): 22 to biological process, 18 to cellular component and 15 to molecular function. Most changes in expression occurred in cytoplasmic membrane-bounded vesicle terms, followed by nucleus, membrane, and plasma membrane in cellular component, which correlated well with the maintenance of floral organ identity and reproductive growth. For the molecular function GO terms, ATP binding and oxidoreductase activity were most representative. Consistently, the biological-process GO terms oxidation–reduction process and metabolic were obviously enriched, and the terms of stress response including response to cadmium ion, cold, heat, karrikin, water deprivation, salt stress, nematode, wounding, and high light intensity were generally detected. This result indicated that most genes involved in cold-induced flowering of *C. goeringii* were associated with various aspects of flower development and stress response.

In addition, we conducted an enrichment analysis using KEGG pathways, which assigned 192 DEGs to 112 pathways. Of these, ‘metabolic pathways’ were the majority, followed by ‘biosynthesis of secondary metabolites’. Details of the pathway annotations for significant hits in unigene sets are provided in Additional file 3: Table S3. Pathways such as Glycolysis/gluconeogenesis, Arginine and proline metabolism, Biosynthesis of amino acids, and Microbial metabolism in diverse environments which respond to flowering physiology and environmental adaptation were also represented, consistent with the obviously enriched biological-process GO terms.

### Differential expression of flowering-related genes

Homology searches of *C. goeringii* floral transcriptome in our previous studies successfully identified a large number of unigenes potentially involved in flowering regulation [5]. These included photoperiodic flowering-related genes *CRY*s, *LHY*, *TOC1*, *FKF1*, *SOC1*, *FT*, *CO-*like; temperature sensitive flowering pathway genes *VIN3*, *VRN2*, and *SVP*; and genes encoding hormone-related GI, Aux/IAA, and other transcription factors such as NAC, bZIP, and bHLH. Of the genes identified previously, at least one member of 21 families exhibited differential expression (Additional file 4: Table S4) and the major changes are listed in Table 3.

**Table 3:** Differentially expressed genes related to floral development. Fold change = log2(expression value of each gene after cold treatment/before cold treatment), expression value = RPKM [total exon reads / mapped reads (millions)] × exon length (kb)

Locus	Ck	Cold	log2(fold_change)	*p*_value	Description
MADS-box gene					
CL8764Contig1	9.8869	0.4959	−4.3174	9.43E-05	SOC1 [*Dendrobium hybrid cultivar*]
CL8650Contig1	20.9806	1.0767	−4.2844	6.47E-05	SOC1 [*Dendrobium hybrid cultivar*]
ep123.comp43097	0.4641	6.4672	2.4787	2.03E-02	SOC1 [*Dendrobium hybrid cultivar*]
ep456.comp50389	4.099	71.4805	4.1242	8.95E-05	SOC1-like protein [*Prunus x yedoensis*]
ep123.comp44547	14.9139	2.9644	−2.3308	6.67E-61	SVP1 [*Actinidia deliciosa*]
ep456.comp28009	114.453	5.2832	−4.4372	0.00E+ 00	MADS-box protein SVP [*Arabidopsis thaliana*]
ep456.comp36141	42.2288	8.5568	−2.3031	4.71E-190	PREDICTED: MADS-box protein SVP [*Vitis vinifera*]
ep456.comp38228	13.3352	0.8606	−3.9538	5.00E-04	FRUITFULL-like MADS box protein 1 [*Dendrobium thyrsiflorum*]
ep456.comp717274	15.2507	232.579	3.9308	1.61E-04	AP1-like MADS-box protein [*Cymbidium ensifolium*]
CL551Contig1	0.0892	5.9554	6.0604	1.87E-06	AGL66 protein [*Eschscholzia californica*]
ep123.comp36586	10.2183	136.628	3.741	3.16E-04	AP3 [*Cymbidium goeringii*]
ep456.comp49009	0.2395	6.6177	4.7883	2.00E-04	AP3 [*Cymbidium goeringii*]
Other flowering and floral related gene					
ep123.comp38616	3.0342	0.5423	−1.9762	9.43E-05	Flowering locus T protein [*Ananas comosus*]
ep456.comp59699	0.8817	0.2078	−2.0139	5.00E-04	Flowering locus D [*Zea mays*]
ep123.comp42797	1.0459	41.3741	5.306	6.47E-05	AP2–7 [*Erycina pusilla*]
ep123.comp218062	26.5682	0.7566	−5.134	2.03E-02	LEAFY [*Cymbidium hybrid cultivar*]
ep123.comp738577	40.6535	1.3573	−4.9045	8.95E-05	LEAFY, partial [*Cattleya hybrid cultivar*]
ep456.comp48048	9.3535	380.927	5.3479	6.67E-61	PREDICTED: late embryogenesis abundant protein D-29-like [*Solanum lycopersicum*]
ep123.comp19364	3.8409	89.0779	4.5356	0.00E+ 00	PREDICTED: late embryogenesis abundant protein D-34-like [*Cicer arietinum*]
CL4811Contig1	0.0451	5.3112	6.8811	4.71E-190	Putative aquaporin TIP5–1 [*Aegilops tauschii*]
CL3697Contig1	1.4658	44.228	4.9152	5.00E-04	PREDICTED: 18 kDa seed maturation protein-like [*Cucumis sativus*]
Plant hormone signaling pathway					
CL8703Contig1	4.2816	0.1327	−5.0116	9.43E-05	PREDICTED: DELLA protein RGL2 [*Vitis vinifera*]
CL9507Contig1	84.2097	4.8002	−4.1328	5.00E-04	PREDICTED: auxin-induced protein 15A-like [*Glycine max*]
ep456.comp45207	0.1394	14.8622	6.736	6.47E-05	SAUR11 - auxin-responsive SAUR family member [*Zea mays*]
ep456.comp58943_c0_seq9:0–1130	0.2642	690.516	11.3521	2.03E-02	ABA related CCAAT-box binding factor HAP5 homolog [*Daucus carota*]
ep123.comp22898	0.4163	25.9618	5.9625	8.95E-05	PREDICTED: ABA related transcription factor GTE1-like [*Vitis vinifera*]
Temperature-responsive factors					
ep456.comp328221	16.1755	6.2463	0.0003	9.43E-05	CBF-like transcription factor [*Trachycarpus fortunei*]
ep123.comp36229	7.1138	−6.1581	0	5.00E-04	Chaperone [*Agave tequilana*]
ep123.comp38271	8.8938	−4.5248	0	6.47E-05	Chaperone [*Agave tequilana*]
ep456.comp54221	11.8107	−3.6707	0.0004	2.03E-02	Chaperone [*Agave tequilana*]
CL5833Contig1	3.8015	−4.1095	0.0001	8.95E-05	Chloroplast small heat shock protein [*Agave americana*]
ep123.comp38941	3.8102	−6.1497	0	6.67E-61	Class-1 LMW heat shock protein [*Ananas comosus*]
ep456.comp63219	89.7919	−4.358	0.0001	4.71E-190	Class-1 LMW heat shock protein [*Ananas comosus*]
CL8847Contig1	18.538	−3.6826	0.0004	5.00E-04	Mitochondrial small heat shock protein [*Capsicum annuum*]
ep123.comp39275	8.2354	−3.6881	0.0004	1.61E-04	PREDICTED: 17.4 kDa class III heat shock protein-like [*Glycine max*]
CL276Contig2	8.1857	−4.9339	0	1.87E-06	PREDICTED: 17.9 kDa class I heat shock protein-like [*Brachypodium distachyon*]
Other transcription factors					
ep456.comp51260	0.2133	208.395	9.9324	9.43E-05	PREDICTED: WRKY transcription factor 6-like [*Vitis vinifera*]
ep123.comp34195	38.4439	2.4205	−3.9894	5.00E-04	PREDICTED: WRKY transcription factor 22 [*Vitis vinifera*]
ep123.comp25957	50.8577	3.9329	−3.6928	6.47E-05	Putative MYB DNA-binding domain superfamily protein [*Zea mays*]
ep456.comp42603	3.1925	364.995	6.837	2.03E-02	Putative R2R3-MYB transcription factor [*Citrus sinensis*]
CL10849Contig1	0.0499	9.6423	7.5943	8.95E-05	TPA: putative MYB DNA-binding domain superfamily protein [*Zea mays*]
ep123.comp101748	11.1484	0.3893	−4.8396	6.67E-61	TPA: putative HLH DNA-binding domain superfamily protein [*Zea mays*]
CL2976Contig1	17.7943	0.9644	−4.2057	0.00E+ 00	Putative bHLH transcription factor [*Arabidopsis thaliana*]
ep456.comp28555	7.4971	−6.2209	0	4.71E-190	CYP86C1 [*Arabidopsis lyrata subsp. lyrata*]

Among the various families, MADS-box family transcription factors, well known for their roles in flower initiation and floral development, were most represented. A total of 12 MADS-box genes showed differential expression – including *CgAP1*, *CgSOC1*, *CgAP3*, and *CgSVP*s – suggesting their potential roles in cold-induced flowering of *C. goeringii*. In addition, there were notable differential expressions among the auxin receptor genes, cytokinin-responsive genes, and *DELLA* family genes, which revealed a critical role of the complex plant hormone network in regulating flower organ primordia formation, organ specification, and final organ development. Most importantly, we found a great number of transcription factors differentially expressed after cold treatment, encoding WRKY, bHLH, MYB, CYP, temperature-responsive HSP and CBF, as well as LATE EMBRYOGENESIS ABUNDANT proteins. These data suggest that accompanying the MADS-box genes, regulators of auxin, gibberellic acid, abscisic acid, and cold signaling were differentially expressed if buds were subjected to cold, highlighting potential involvement of these regulators in *C. goeringii* flowering competence.

### Validation of gene expression in response to cold

**Fig. 4:**
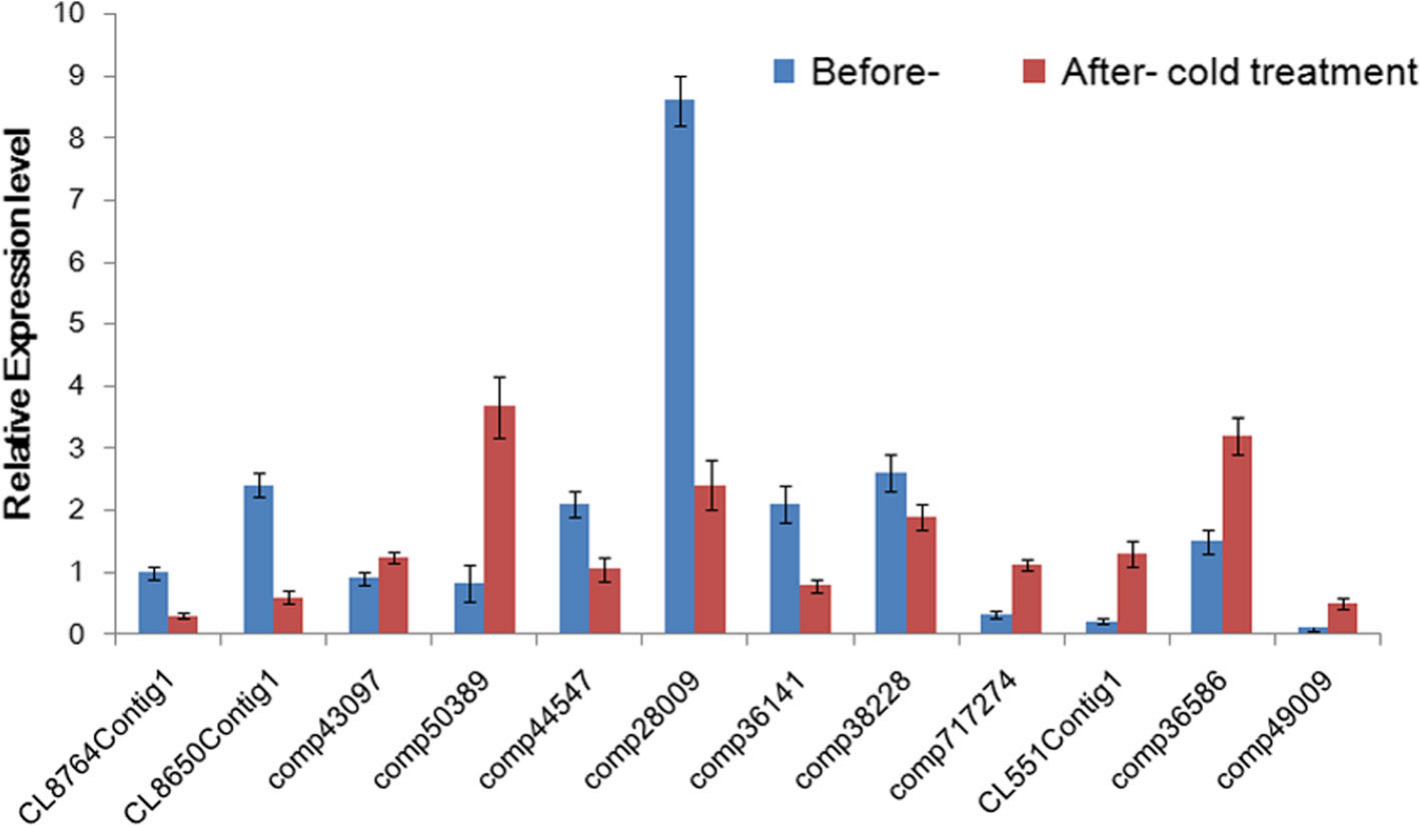
The quantitative RT-PCR analysis of gene expression before and after cold treatment The y-axis indicates fold change in expression among the samples. Expression levels were normalized using the threshold cycle values obtained for the *Ubiquitin* and *Actin* genes. Error bars indicate the standard deviation of the mean (SD) (*n* = 3). Three replicates were analyzed, with similar results. One way ANOVA with Bonferroni multiple comparison test significant at *P* < 0.05 between the two samples

The digital gene expression analysis showed that MADS-box genes represented the majority of gene families that were differentially expressed. To validate the DEG results and determine the potential roles of the MADS-box genes referred to above, we obtained their full length sequences and confirmed their expression responses to cold using qRT-PCR. EST-encoding ubiquitin and actin genes were used as internal controls, to which gene expression was normalized. The same mRNA flower samples used for transcriptome sequencing served as templates. Among the 12 genes tested, 11 showed a significant expression difference after cold treatment, with six down-regulated and five up-regulated. Among them, three paralogs of *SVP* genes ep123.comp44547, ep456.comp28009, and ep456.comp36141 had the most significant repressed expression, with decreases in the range of 50–70%. In contrast, transcripts of *SOC1*-like ep456.comp50389, *AP3*-like ep123.comp36586, ep456.comp49009, and AGL66 clade CL551Contig1 increased by 2–8 fold, identical to those obtained by DEG expression profiling (Fig. 4).

### *CgSVP* expression correlates with cold-regulated flowering of *C. goeringii*

**Fig. 5:**
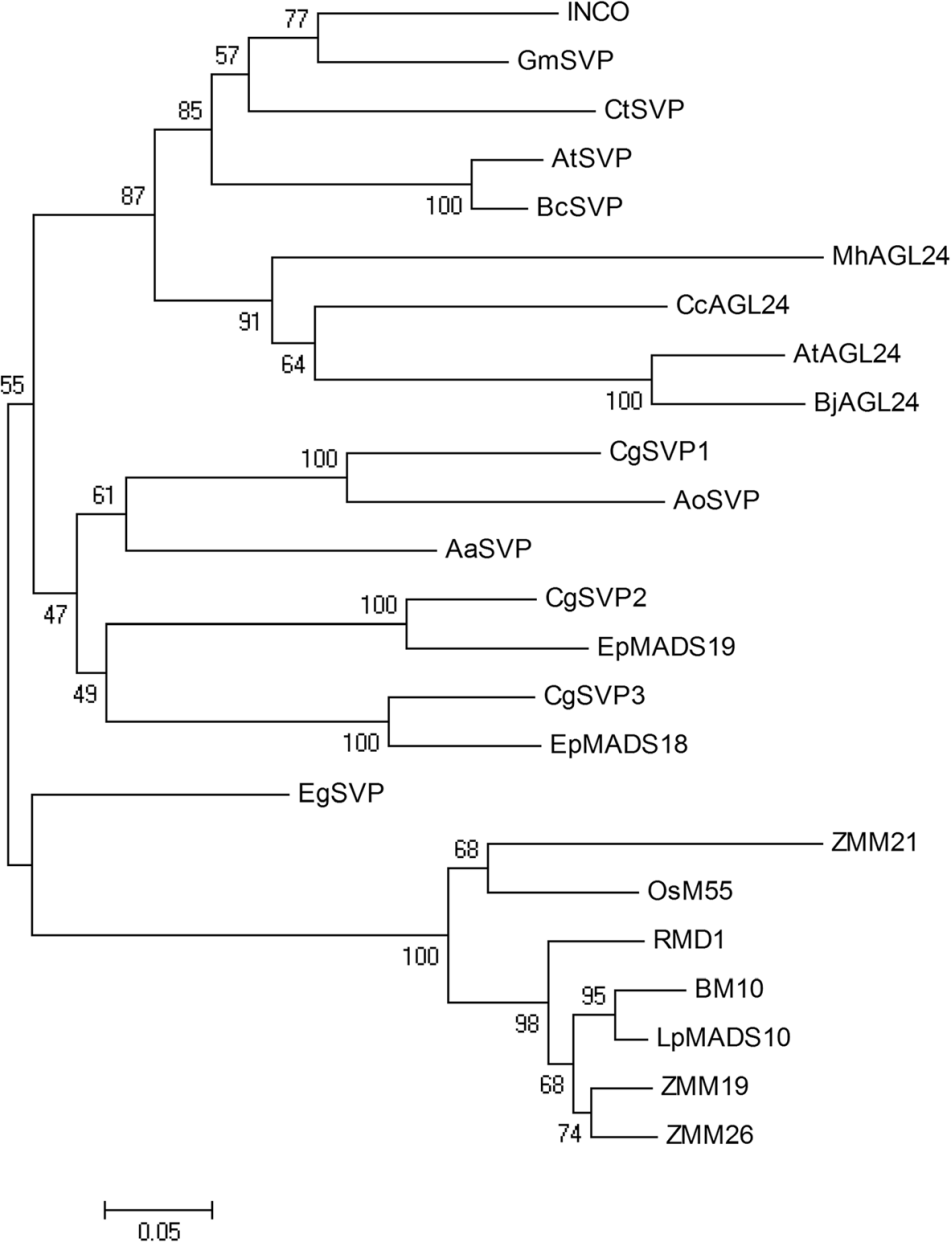
Phylogenetic analysis of the SVP-like proteins from different plant species. Amino acid sequences were aligned by the ClustalW 2.0, and phylogenetic relationships were reconstructed using a maximum-likelihood (ML) method in PHYML software with JTT amino acid substitution model. Bootstrap values for 1000 replicates were used to assess the robustness of the trees. Previously published plant SVP protein sequences were retrieved from GenBank database. Aa: *Anthurium amnicola*, Ao: *Apostasia odorata*, Bj: *Brassica juncea*, Br: *Brassica rapa*, Cc: *Carya cathayensis*, Ct: *Citrus trifoliate*, Eg, *Elaeis guineensis*, Ep: *Erycina pusilla*, Gm:*Glycine max*, Lp: *Lolium perenne*, Mh: *Monotropa hypopitys*, Os, *Oryza sativa*.(AtSVP: BAD43004, BM10: ABM21529, LpMADS10: AAZ17549, ZMM19: NP_001105148, ZMM26: NP_001105154, ZMM21: CAD23411, OsM55: BAD35842, RMD1: BAA81880, INCO: CAG27846, BrSVP: XP_009112514, EgSVP: XP_010942683, AaSVP: JAT54145, GmSVP: NP_001240951, AoSVP: AIZ95422, CtSVP: ACJ09170, EpMADS18: AJB29196, EpMADS19: AJB29197, MhAGL24: AQM52285, CcAGL24: AHI85951, AtAGL24: OAO97218, BjAGL24: AFM77904)

**Fig. 6:**
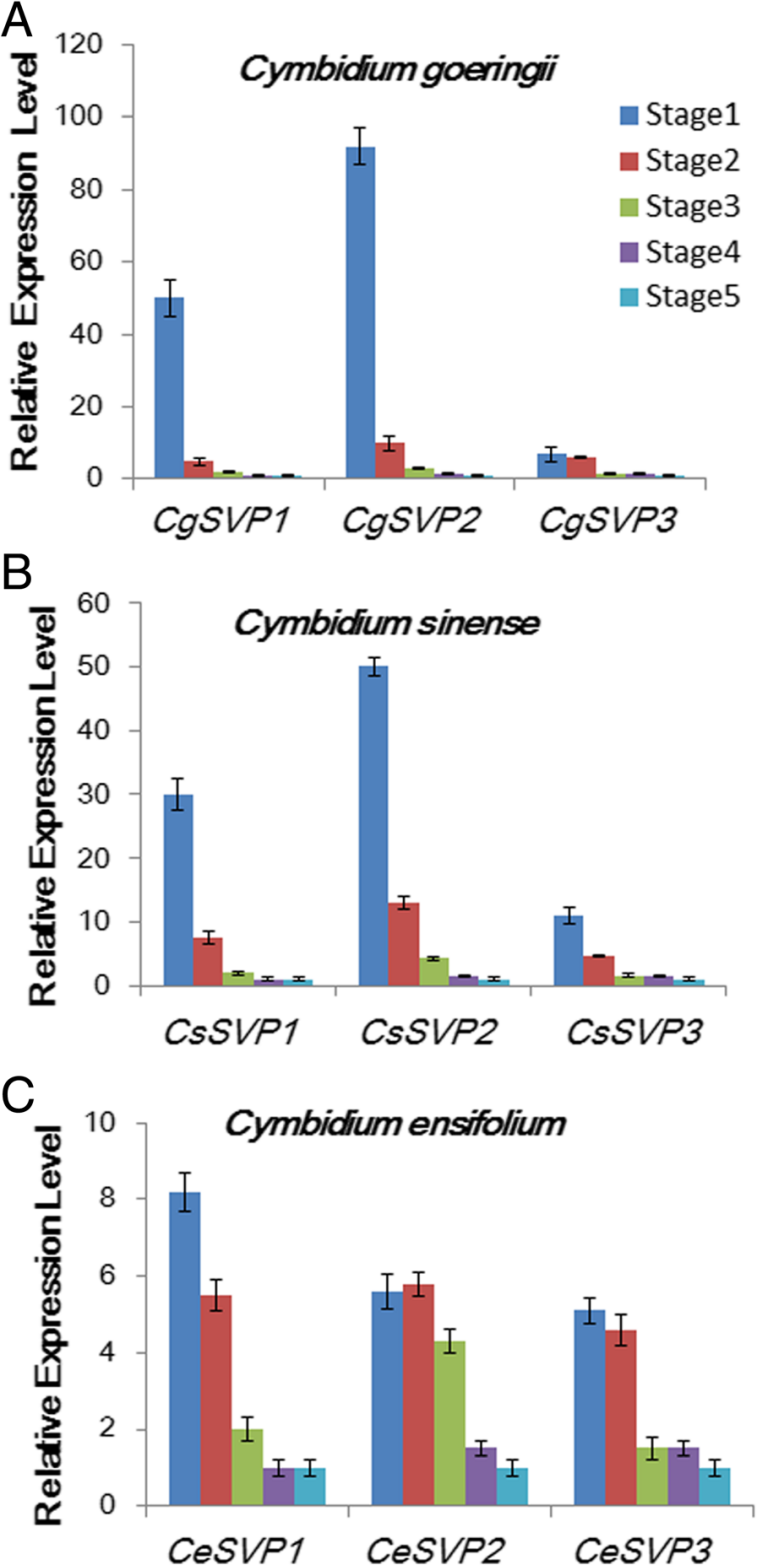
Expression patterns of *SVP* genes in different cultivated varieties of Cymbidium genus (a-c).. The y-axis indicates fold change in expression among the floral buds at different developmental stages. Expression levels were normalized using the threshold cycle values obtained for the *Ubiquitin* and *Actin* genes. Error bars indicate the standard deviation of the mean (SD) (*n* = 3). Three replicates were analyzed, with similar results

The *SVP* genes showed the most significant decrease after cold treatment, implying their potential important roles in responding to cold during the process of flower development. Phylogenetic analysis showed that the *SVP* homologs of orchid plants form a separate clade and divided into three sub-clades. As shown in Fig. 5, ep456.comp28009, ep123.comp44547, and ep456.comp36141 showed high similarity with *Apostasia odorata SVP*, *Erycina pusilla MADS19*, and *Erycina pusilla MADS18*, respectively. We correspondingly named them *CgSVP1*, *CgSVP2*, and *CgSVP3* belonging to three different sub-clades (Fig. 5). Accumulation profiles in the process of floral development indicated that *CgSVP1*, *CgSVP2*, and *CgSVP3* accumulated in stage 1, at levels 52, 98, and 8.5-fold higher than those in stage 5, respectively. With maturing of the flower, their expressions gradually decreased, suggesting a negative relationship with flower development (Fig. 6a). Moreover, we found these expression patterns of *SVP* genes altered in different cultivated varieties of *Cymbidium* spp. with a range of flowering habits. For example, in *C. sinense*, which is a close relative of *C. goeringii* and also flowers in winter, *CsSVP* genes dramatically accumulated in stage 1, and decreased during bud development stages 1–5 from 100- to 10-fold (Fig. 6b, Additional file 5: Figure S1). In contrast, *C. ensifolium*, which flowers in summer and needs no cold treatment, the *CeSVP* genes showed no more than 8-fold change during floral development (Fig. 6c, Additional file 6: Figure S2).

**Fig. 7:**
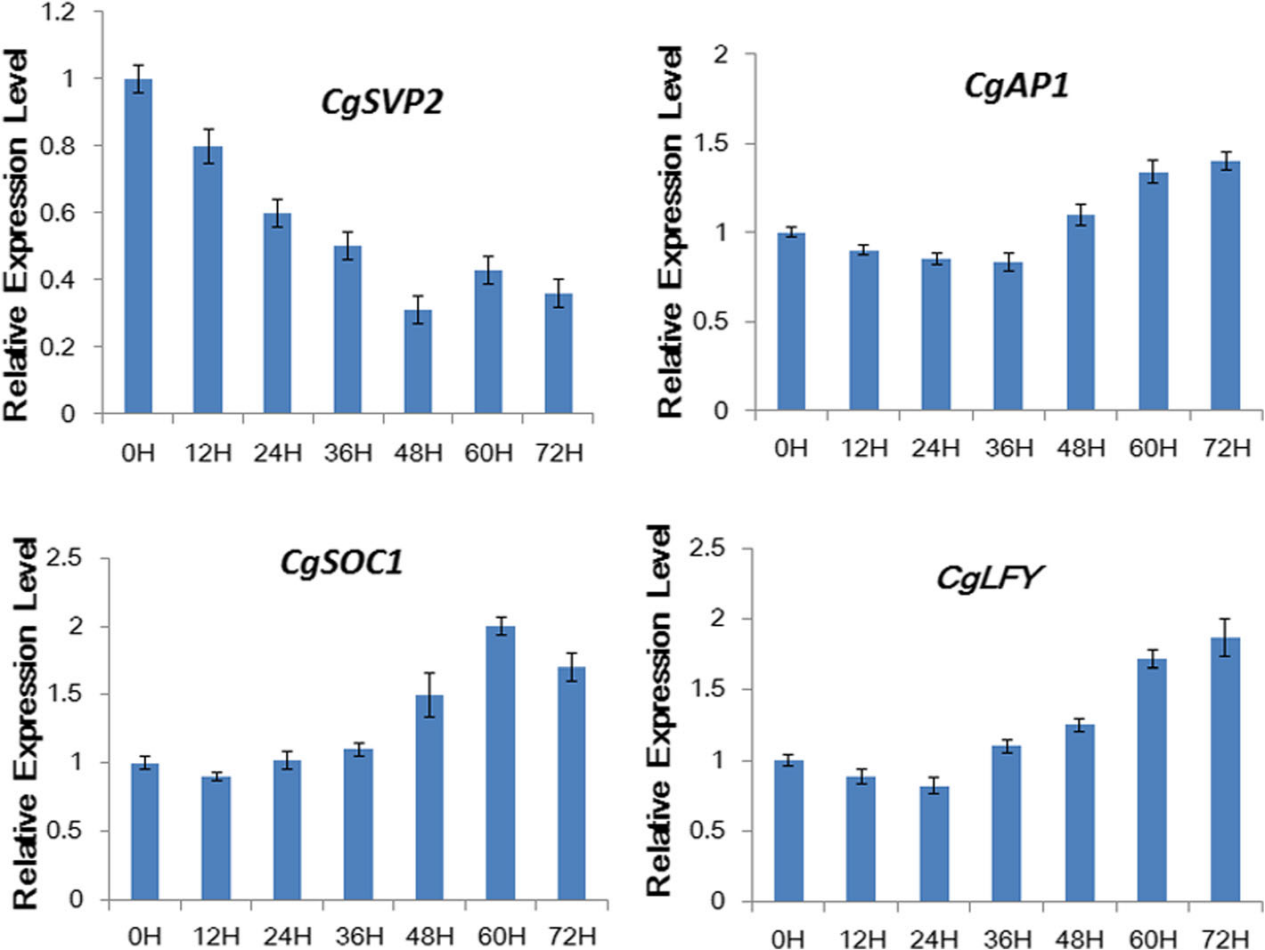
Time course cold response of *CgSVP2* and floral pathway integrators. The y-axis indicates fold change in expression among the samples. Expression levels were normalized using the threshold cycle values obtained for the *Ubiquitin* and *Actin* genes. Error bars indicate the standard deviation of the mean (SD) (n = 3). Three replicates were analyzed, with similar results

Moreover, a time-course responses of Cg*SVP* and flowering integrators *CgAP1*, *CgFT*, *CgSOC1*, *CgLFY* to low temperature were determined, to further validate the *CgSVP* transcript associated with cold response and *C. goeringii* flowering behavior. *CgSVP2* which showed the largest decrease in expression in cold condition was chosen. The Result as shown in Fig. 7 indicated that the expression of *CgSVP2* began to decrease after the 12-h treatment at low temperature. However, expression levels of *CgSOC1*, *CgLFY*, and *CgAP1* increased after 24–36 h of low-temperature treatment. These results suggested that the *CgSVP* genes showed population and cultivar variation in expression that correlated with cold-regulated flowering and probably function in the early stage of low temperature-induced flowering of *C. goeringii.*

**Fig. 8:**
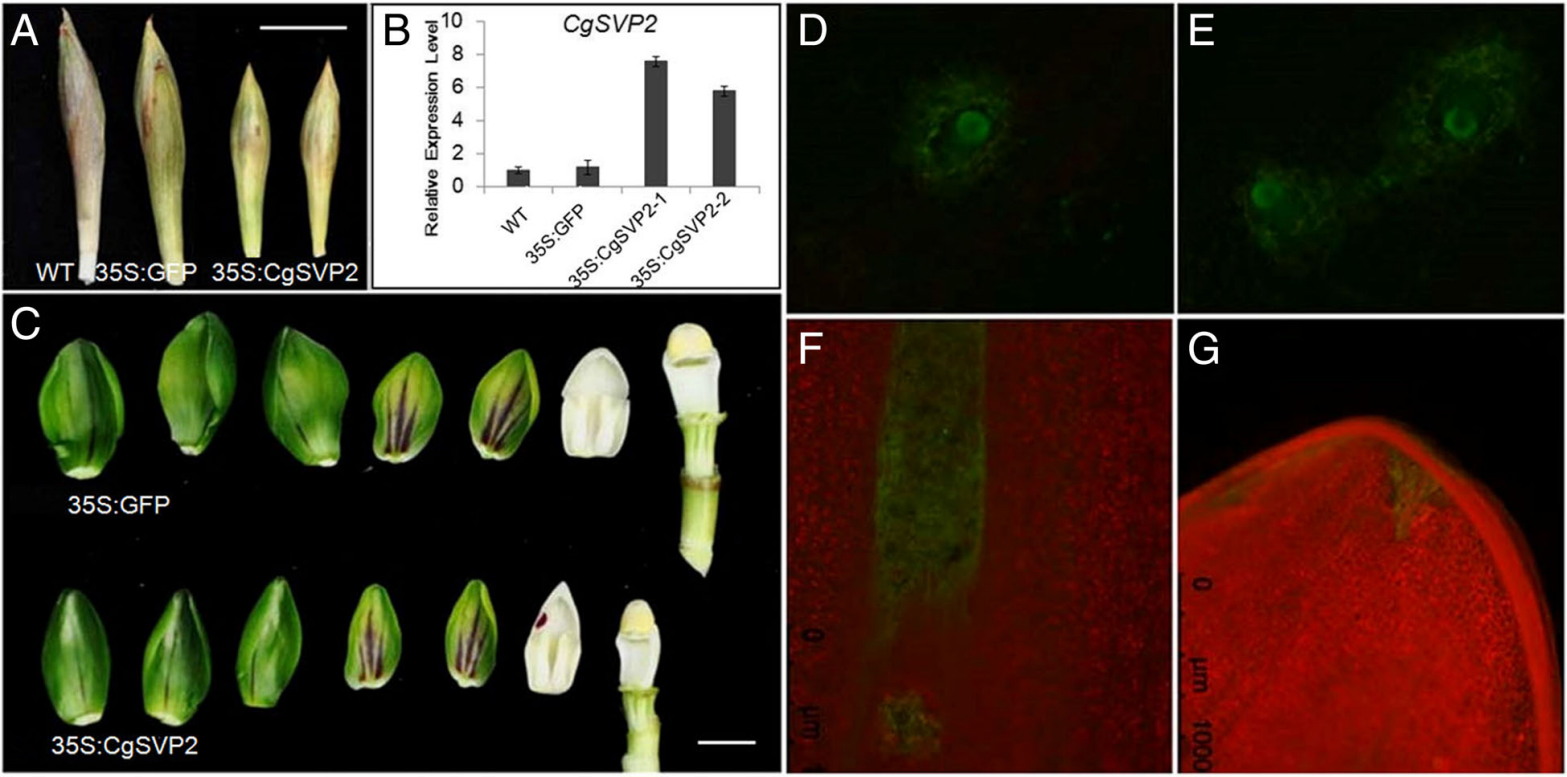
Transient over-expression of *CgSVP2* inhibits flower bud development. **a** Flower bud of wild-type (WT), *35S:eGFP* and *35S:CgSVP2.* Photos were taken in 3 weeks after injection of *Agrobacterium*. **b** Increased expression level of *CgSVP2* in the flower bud infiltrated with *35S:CgSVP2*, **c** Appearance of floral organs in the flower bud infiltrated with *35S:CgSVP2* and *35S:eGFP*. D-G: GFP signal detected after injection of *Agrobacterium* for 3 days (**d**), 5 days (**e**), 3 weeks (**f**), and 5 weeks (**g**) as a control to confirm continuous gene expression

### Transient over-expression of *CgSVP* in *C. goeringii* floral bud

In order to verify the function of *CgSVP* genes in vivo, we carried out a transient expression experiment in the floral bud of *C. goeringii*. *CgSVP2* which showed the strongest changes in gene expression during floral development and in response to cold was chosen for function analysis. *Agrobacterium* carrying *35S:CgSVP2* or *35S:eGFP* was injected into the flower buds (60 days after floral initiation) of *C. goeringii*. After 3 weeks of growth in the condition of 25/10 °C and photoperiod of 16/8 h, we observed a retarded growth of flower buds injected with *CgSVP2*, which showed 16–21% decrease in length and 10–14% decrease in width. Meanwhile, floral organ size is also remarkably repressed, especially for the differentiation of the lip and the column compared with the control group injected *35S:eGFP* (Fig. 8). Together with functional studies of *SVP-like* genes in other species, these data are consistent with *SVP* genes being repressors of flower bud development that have been coopted for cold-induced *C. goeringii* flowering.

### Protein–protein interactions

**Fig. 9:**
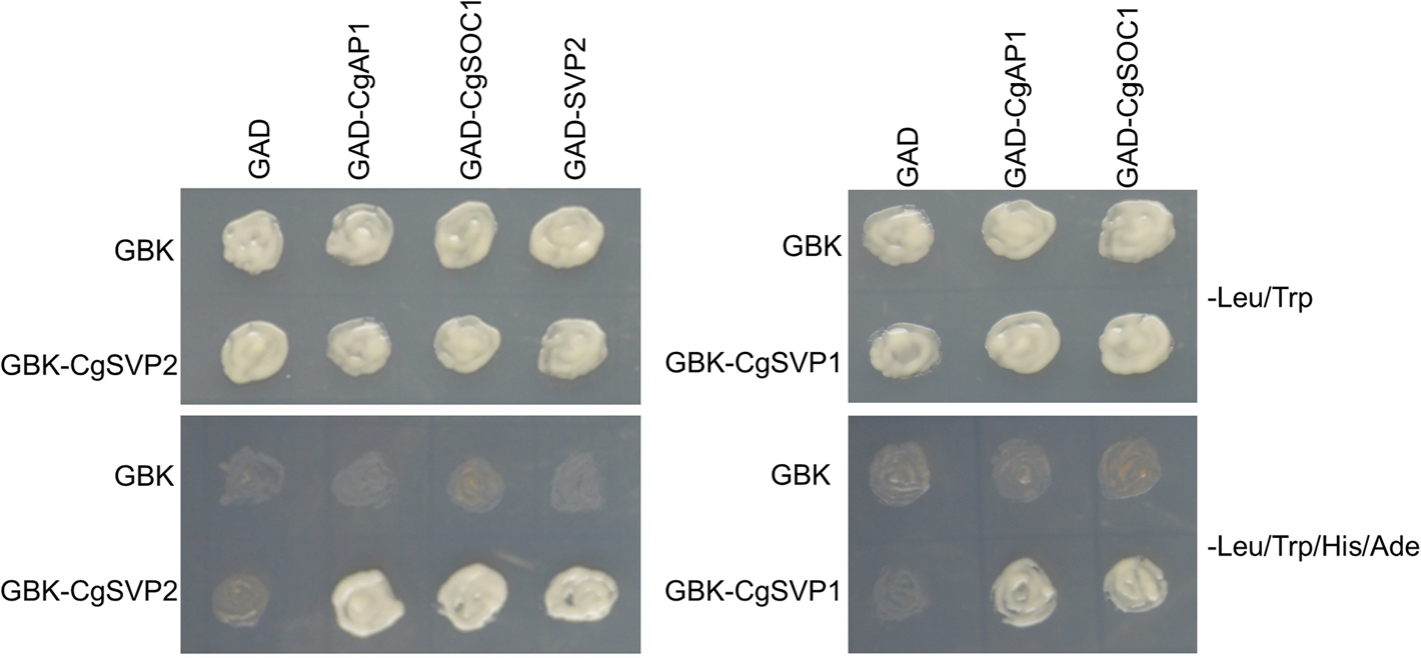
Protein–protein interaction among CgAP1, CgSOC1 and CgSVP proteins. Protein interaction behavior is indicated by growth on selection medium lacking leucine, tryptophan, histidine, and adenine

It is well known that most MADS-box proteins function in the form of homodimers and heterodimers [33, 34]. Considering the orchid-specific whole-genome duplication event which provided extensive genes for neo-functionalization, sub-functionalization, or dosage strengthening [1, 35], we used yeast two-hybrid assays to identify the interacting partner of CgSVP proteins. The full-length coding region of *CgSVP1, CgSVP2*, and *CgSVP3* were used as the bait to screen the cDNA library of *C. goeringii* floral buds, and 87 positive clones were obtained. Sequencing analyses suggested that 11 of them encoded CgSOC1, CgAP1, and CgSVP2, respectively. To further confirm the protein interactions, the full-length coding regions of *CgSOC1* and *CgAP1* were cloned, after which the interaction between the encoded proteins and CgSVP proteins was confirmed in yeast cells. As shown in Fig. 9, CgSVP2 could homodimerize as well as heterodimerize with CgAP1 and CgSOC1, and interactions also exist between CgSVP1-CgAP1 and CgSVP1-CgSOC1. However, we can neither detect protein-protein interactions between CgSVP3 and CgAP1/CgSOC1 nor protein homodimerization activities of CgSVP1 and CgSVP3 (Additional file 7: Figure S3). Distinct protein interaction patterns suggest that *CgSVP* genes may perform conserved, but distinct functions similarly to *SVP-like* genes from other species which showed conservation and divergence of biological roles in floral meristem identity.

## Discussion

### Cold-regulated floral development of *C. goeringii*

The correct timing for flowering is critical in plant adaptation and survival in perennials [36]. After an active growth period in summer, specific photoperiod and/or temperature conditions are determinant environmental cues for bud set, growth cessation, and following release. Vegetative growth of *C*. *goeringii* dominates during summer–autumn, when the floral transition begins in the newly developed axillary buds under long photoperiods and warm conditions. Then, the initiated floral buds grow very slowly and a 5–10 °C cold condition is required in winter to promote floral development and finally blooming during January–March.

In this study, comparative transcriptome analyses showed most obvious enrichment for metabolism and related gene functions within biological process. KEGG pathways related to metabolism such as glycolysis/gluconeogenesis, arginine, proline, and microbial metabolisms were also representative, consistent with previous reports showing extensive reprogramming of transcriptional and metabolic pathways involved in seasonal dormancy of perennial plants [37].

Moreover, 21 gene families related to flowering and floral development exhibited differential expression, which included flowering integrator factors FT, FD, and LFY proteins and the well-known MADS-box *AP1*, *SOC1*, *AP3*, and *SVP* homologs, as well as many other regulatory transcription factors involved in plant hormone signal transduction pathways. Most importantly, regarding the DEG data, we found significant changes in the regulators that contributed to reproductive organ development, such as pollen related aquaporin *TIP5–1* (CL4811Contig1), seed maturation protein-like (CL3697Contig1), which indicated a cold response of reproductive organs [38].

Results also highlighted that genes responding to cadmium ion, cold, heat, karrikin, water deprivation, salt stress, nematode, wounding, and high light intensity were involved in the flowering process. Among them, we noted a higher level of cold-response *CBF* in the cold condition; interestingly, a great number of heat shock proteins and chaperones showed dominant expression level in normal growth conditions, and marked decreases after cold treatment. Small heat shock proteins form the first line of defense against protein aggregation in order to maintain protein homeostasis and minimize injury when exposed to a harsh environment [39–41]. As mentioned above, *C. goeringii* only bloomed following exposure to cold. Otherwise, the buds withered in our field experiment, consistent with the DEG data showing a heat shock stress response under natural conditions. This result indicated that heat shock stress was the primary reason for failure to bloom.

### Cold-responsive gene expression of *CgSVP*s in *C. goeringii*

The role of *SVP* genes in the temperature-dependent control of flowering in annual species and bud dormancy in perennial species have been widely studied, and appear to be functionally diversified in regulating flowering time. In addition, the expression of *CgSVP* genes in different cultivated varieties of *Cymbidium* spp. with different flowering habits fulfilled our predictions for a gene directly or indirectly involved in flowering differences between the populations. To further validate if the *CgSVP* transcript associated with cold response and *C. goeringii* flowering behavior, we used qRT-PCR to determine the time-course response of Cg*SVP*2 and flowering regulatory genes *CgAP1*, *CgFT*, *CgSOC1*, and *CgLFY* to low temperature, according to the transcriptome sequencing. We found that the response of *CgSVP*s to low temperature was earlier than that of *CgSOC1*, *CgLFY*, and *CgAP1*, which is very similar to the response of *FLC* before *AP1* to vernalization. Assuming they are indeed repressors, we propose that *CgSVP* genes play an important role upstream of *CgSOC1*, *CgLFY*, and *CgAP1*.

### Homodimerization and Heterodimerization of SVP function in flowering regulation

Considerable effort has been made to identify and characterize SVP genes in many monocots and dicots, and has demonstrated similar molecular activity and growth inhibitory function among *SVP* genes, but differential functional pathways have been found for individual *SVP* gene family members. For example, the dicot *Arabidopsis* SVP protein forms a complex with related temperature-responsive proteins FLC and FLM. In monocot wheat, TaSVP protein regulates the flowering activator TaVRN1 by directly binding to the CArG box located in the promoter of *TaVRN1* gene, and can also homodimerize and form heterodimers with several transcription factors involved in flowering control and vernalization [22]. The observed expression pattern of *CgSVP* genes that correlated with cold-regulated flowering led us to hypothesize that decreased expression of *CgSVP* genes during cold days negates the repressive effects on floral growth of *C. goeringii*. However, their exact roles in the flowering pathway remain unclear. Our yeast two-hybrid assay suggested that CgSVP2 interacts with CgSOC1 and CgAP1, similar to reports in petunia [42], rice [43], *Antirrhinum*, and *Arabidopsis* [44], implying that protein–protein interactions and formation of complexes are the basis of MADS-box transcription factor function. Therefore, the interaction between members of these clades may represent an evolutionarily conserved property that is important for their function, and AP1 probably serves as a hub between the interacting proteins of the flower induction pathway (such as SVP, SOC1, and AGL24) and the floral organ identity proteins [33]. However, this might not represent a general property for homodimerization of SVP proteins, since we failed to detected homodimerization of CgSVP1 and CgSVP3, and dicot AtSVP also does not homodimerize [44]. This suggests that there will be differences among the interactions of transcriptional activator/repressor MADS-box proteins that regulate flowering time in plants. Overall, the physical associations of SVP with other partners further our knowledge of the regulation of flowering in low-temperature-sensitive orchids, which could offer clues for further studies to determine their functions in flowering regulation.

## Conclusions

As a horticulturally important ornamental plant in the orchid family, *C. goeringii* is typically characterized by its winter-blooming behavior. To better understand the molecular regulation of *C. goeringii* winter-blooming, we study the process of floral bud development by cytobiology observations and physiological experiments, and compared the transcriptome of floral bud before and after cold-treatment. Consequently, 582 candidate genes involved in metabolic process, flowering time, hormone signaling, stress response, and cell cycle, were found to be related to the process of low-temperature-induced flowering of *C. goeringii*. Based on full-length cDNA sequence analysis and expression validation, we found that three genes within the SHORT VEGETATIVE PHASE (SVP) sub-group of MADS-box gene family are most closely related with low-temperature-induced flowering and interact with CgAP1 and CgSOC1, suggesting that they may synergistically control the process of *C. goeringii* flowering in winter. This work represents the first exploration of flowering physiology of *C. goeringii,* and provides gene expression information that could facilitate our understanding of molecular regulation of orchid plant winter-flowering.

## Additional files

Additional file 1: **Table S1.** The primers used for Realtime RT-PCR. (XLSX 11 kb)

Additional file 2: **Table S2.** List of gene ID and transcript fold changes of the all DEGs (differentially expressed genes). (XLSX 47 kb)

Additional file 3: **Table S3.** Details of the pathway annotations for the DEGs by KEGG. (XLSX 18 kb)

Additional file 4: **Table S4.** Function annotations of the DEGs after cold treatment. (XLSX 39 kb)

Additional file 5: **Figure S1.** Floral development stages of *Cymbidium sinense. (TIF 3245 kb)*

Additional file 6: **Figure S2.** Floral development stages of *Cymbidium ensifolium. (TIF 3232 kb)*

Additional file 7: **Figure S3.** Homodimerization and Heterodimerization of *Cymbidium goeringii* MADS-box proteins. (JPG 142 kb)

## Abbreviations

AGL: AGAMOUS-Like
AP1: APETALA1
AP2/ERF: APETALA2/Ethylene Responsive Factor
AP3: APETALA3
ARF: Auxin response factor
Aux/IAA: Auxin/indole-3-acetic acid
bHLH: basic helix-loop-helix
bZIP: Basic leucine zipper
CBF: C-repeat binding factor
CO: CONSTANS
CYP: Cytochromes P450
DAM: Dormancy-associated MADS-BOX
DEGs: Differentially expressed genes
FLC: Flowering Locus C
FT: Flowering Locus T
GI: GA insensitive
GO: Gene ontology
HSP: Heat shock protein
KEGG: Kyoto encyclopedia of genes and genomes
LFY: Leafy
LHY: Late elongated hypocotyl
MYB: Myeloblastosis transcription factors
NAC: NAM, ATAF, and CUC transcription factor
RPM: Reads per million
SAM: Shoot apical meristem
SAUR: Small auxin up RNA
SOC: Suppressor of CONSTANS
SVP: Short vegetative phase
TIP: Tonoplast intrinsic protein
TOC: Timing of CAB expression
VIN: Vernalization insensitive
VRN: Vernalization

## References

[1] Cai J, Liu X, Vanneste K, Proost S, Tsai WC, Liu KW (2015). The genome sequence of the orchid *Phalaenopsis equestris* (vol 47, pg 65, 2015). Nat Genet.

[2] Wong DCJ, Pichersky E, Peakall R. The biosynthesis of unusual floral volatiles and blends involved in orchid pollination by deception: current Progress and future prospects. Front Plant Sci. 2017;8:1955. 10.3389/fpls.2017.01955 PMC5693887 29181016

[3] Du P, Cribb P. The genus Cymbidium. London and Portland, Oregon: Christopher Helm and Timber Press. 1988.

[4] Li XB, Xiang L, Wang Y, Luo J, Wu C, Sun CB (2014). Genetic diversity, population structure, pollen morphology and cross-compatibility among Chinese *Cymbidiums*. Plant Breed.

[5] Yang FX, Zhu GF, Wang Z, Liu HL, Xu QQ, Huang D, et al. Integrated mRNA and microRNA transcriptome variations in the multi-tepal mutant provide insights into the floral patterning of the orchid *Cymbidium goeringii*. BMC Genomics. 2017;18(1):367. 10.1186/s12864-017-3756-9 PMC5426072 28490318

[6] Liu XF, Huang Y, Li F, Xu CJ, Chen KS (2014). Genetic diversity of 129 spring orchid (cymbidium goeringii) cultivars and its relationship to horticultural types as assessed by EST-SSR markers. Sci Hortic-Amsterdam.

[7] Xiang L, Chen Y, Chen L, Fu X, Zhao K, Zhang J, Sun C. B and E MADS-box genes determine the perianth formation in *Cymbidium goeringii* Rchb.f. Physiol Plant. 2017. 10.1111/ppl.12647. 10.1111/ppl.12647 28967227

[8] Rosas U, Mei Y, Xie Q, Banta JA, Zhou RW, Seufferheld G (2014). Variation in Arabidopsis flowering time associated with cis-regulatory variation in CONSTANS. Nat Commun.

[9] Putterill J, Varkonyi-Gasic E (2016). FT and florigen long-distance flowering control in plants. Curr Opin Plant Biol.

[10] Hori K, Matsubara K, Yano M (2016). Genetic control of flowering time in rice: integration of Mendelian genetics and genomics. Theor Appl Genet.

[11] Krasileva KV, Vasquez-Gross HA, Howell T, Bailey P, Paraiso F, Clissold L (2017). Uncovering hidden variation in polyploid wheat. P Natl Acad Sci USA.

[12] Sharma N, Ruelens P, D'hauw M, Maggen T, Dochy N, Torfs S (2017). A flowering locus C homolog is a Vernalization-regulated repressor in *Brachypodium* and is cold regulated in wheat. Plant Physiol.

[13] Brambilla V, Gomez-Ariza J, Cerise M, Fornara F (2017). The importance of being on time: regulatory networks controlling photoperiodic flowering in cereals. Front Plant Sci.

[14] Ding J, Nilsson O (2016). Molecular regulation of phenology in trees-because the seasons they are a-changin. Curr Opin Plant Biol.

[15] Yordanov YS, Ma C, Strauss SH, Busov VB (2014). EARLY BUD-BREAK 1 (EBB1) is a regulator of release from seasonal dormancy in poplar trees. P Natl Acad Sci USA.

[16] Wisniewski M, Norelli J, Artlip T (2015). Overexpression of a peach CBF gene in apple: a model for understanding the integration of growth, dormancy, and cold hardiness in woody plants. Front Plant Sci.

[17] Fujiwara S, Oda A, Yoshida R, Niinuma K, Miyata K, Tomozoe Y (2008). Circadian clock proteins LHY and CCA1 regulate SVP protein accumulation to control flowering in Arabidopsis. Plant Cell.

[18] Gregis V, Sessa A, Dorca-Fornell C, Kater MM (2009). The Arabidopsis floral meristem identity genes AP1, AGL24 and SVP directly repress class B and C floral homeotic genes. Plant J.

[19] Tao Z, Shen L, Liu C, Liu L, Yan Y, Yu H (2012). Genome-wide identification of SOC1 and SVP targets during the floral transition in Arabidopsis. Plant J.

[20] Trevaskis B, Tadege M, Hemming MN, Peacock WJ, Dennis ES, Sheldon C (2007). Short vegetative phase-like MADS-box genes inhibit floral meristem identity in barley. Plant Physiol.

[21] Kane NA, Agharbaoui Z, Diallo AO, Adam H, Tominaga Y, Ouellet F (2007). TaVRT2 represses transcription of the wheat vernalization gene TaVRN1. Plant J.

[22] Kane NA, Danyluk J, Tardif G, Ouellet F, Laliberté J-F, Limin AE (2005). TaVRT-2, a member of the StMADS-11 clade of flowering repressors, is regulated by vernalization and photoperiod in wheat. Plant Physiol.

[23] Li Z, Reighard GL, Abbott AG, Bielenberg DG (2009). Dormancy-associated MADS genes from the EVG locus of peach [*Prunus persica* (L.) Batsch] have distinct seasonal and photoperiodic expression patterns. J Exp Bot.

[24] Wells CE, Vendramin E, Jimenez Tarodo S, Verde I, Bielenberg DG (2015). A genome-wide analysis of MADS-box genes in peach [*Prunus persica* (L.) Batsch]. BMC Plant Biol.

[25] Kitamura Y, Takeuchi T, Yamane H, Tao R (2016). Simultaneous down-regulation of DORMANCY-ASSOCIATED MADS-box6 and SOC1 during dormancy release in Japanese apricot (*Prunus mume*) flower buds. J Hrtic Sci Biotech.

[26] Wu R, Wang T, McGie T, Voogd C, Allan AC, Hellens RP (2014). Overexpression of the kiwifruit SVP3 gene affects reproductive development and suppresses anthocyanin biosynthesis in petals, but has no effect on vegetative growth, dormancy, or flowering time. J Exp Bot.

[27] Wu R, Wang T, Warren BAW, Allan AC, Macknight RC, Varkonyi-Gasic E (2017). Kiwifruit SVP2 gene prevents premature budbreak during dormancy. J Exp Bot.

[28] Yang FX, Zhu GF. Digital gene expression analysis based on de novo transcriptome assembly reveals new genes associated with floral organ differentiation of the orchid plant *Cymbidium ensifolium*. PLoS One. 2015;10(11):e0142434. 10.1371/journal.pone.0142434 PMC4651537 26580566

[29] Wang Y, Huang H, Ma YP, Fu JX, Wang LL, Dai SL (2014). Construction and de novo characterization of a transcriptome of *Chrysanthemum lavandulifolium*: analysis of gene expression patterns in floral bud emergence. Plant Cell Tiss Org.

[30] Conesa A, Gotz S, Garcia-Gomez JM, Terol J, Talon M, Robles M (2005). Blast2GO: a universal tool for annotation, visualization and analysis in functional genomics research. Bioinformatics.

[31] Kanehisa M, Goto S (2000). KEGG: kyoto encyclopedia of genes and genomes. Nucleic Acids Res.

[32] Wroblewski T, Tomczak A, Michelmore R (2010). Optimization of *Agrobacterium*-mediated transient assays of gene expression in lettuce, tomato and *Arabidopsis*. Plant Biotechnol J.

[33] Honma T, Goto K (2001). Complexes of MADS-box proteins are sufficient to convert leaves into floral organs. Nature.

[34] de Folter S, Immink RGH, Kieffer M, Parenicova L, Henz SR, Weigel D (2005). Comprehensive interaction map of the Arabidopsis MADS box transcription factors. Plant Cell.

[35] Zhang GQ, Liu KW, Li Z, Lohaus R, Hsiao YY, Niu SC, Wang JY, Lin YC, Xu Q, Chen LJ (2017). The *Apostasia* genome and the evolution of orchids. Nature.

[36] Friedman J (2017). Variation in gene regulation underlying annual and perennial flowering in Arabideae species. Mol Ecol.

[37] Rohde A, Bhalerao RP (2007). Plant dormancy in the perennial context. Trends Plant Sci.

[38] Soto G, Fox R, Ayub N, Alleva K, Guaimas F, Erijman EJ (2010). TIP5;1 is an aquaporin specifically targeted to pollen mitochondria and is probably involved in nitrogen remobilization in *Arabidopsis thaliana*. Plant J.

[39] Haslbeck M, Vierling E (2015). A first line of stress defense: small heat shock proteins and their function in protein homeostasis. J Mol Biol.

[40] Ohama N, Sato H, Shinozaki K, Yamaguchi-Shinozaki K (2017). Transcriptional regulatory network of plant heat stress response. Trends Plant Sci.

[41] Jacob P, Hirt H, Bendahmane A (2017). The heat-shock protein/chaperone network and multiple stress resistance. Plant Biotechnol J.

[42] Immink RGH, Ferrario S, Busscher-Lange J, Kooiker M, Busscher M, Angenent GC (2003). Analysis of the petunia MADS-box transcription factor family. Mol Gen Genomics.

[43] Fornara F, Parenicova L, Falasca G, Pelucchi N, Masiero S, Ciannamea S (2004). Functional characterization of OsMADS18, a member of the AP1/SQUA subfamily of MADS box genes. Plant Physiol.

[44] Masiero S, Li MA, Will I, Hartmann U, Saedler H, Huijser P (2004). INCOMPOSITA: a MADS-box gene controlling prophyll development and floral meristem identity in antirrhinum. Development.

