# Supplementary figures and images for: Low-temperature-induced changes in the transcriptome reveal a major role of CgSVP genes in regulating flowering of Cymbidium goeringii
Source: BMC Genomics. 2019 Jan 17;20:53. doi: 10.1186/s12864-019-5425-7 (PMC6335714; doi:10.1186/s12864-019-5425-7)

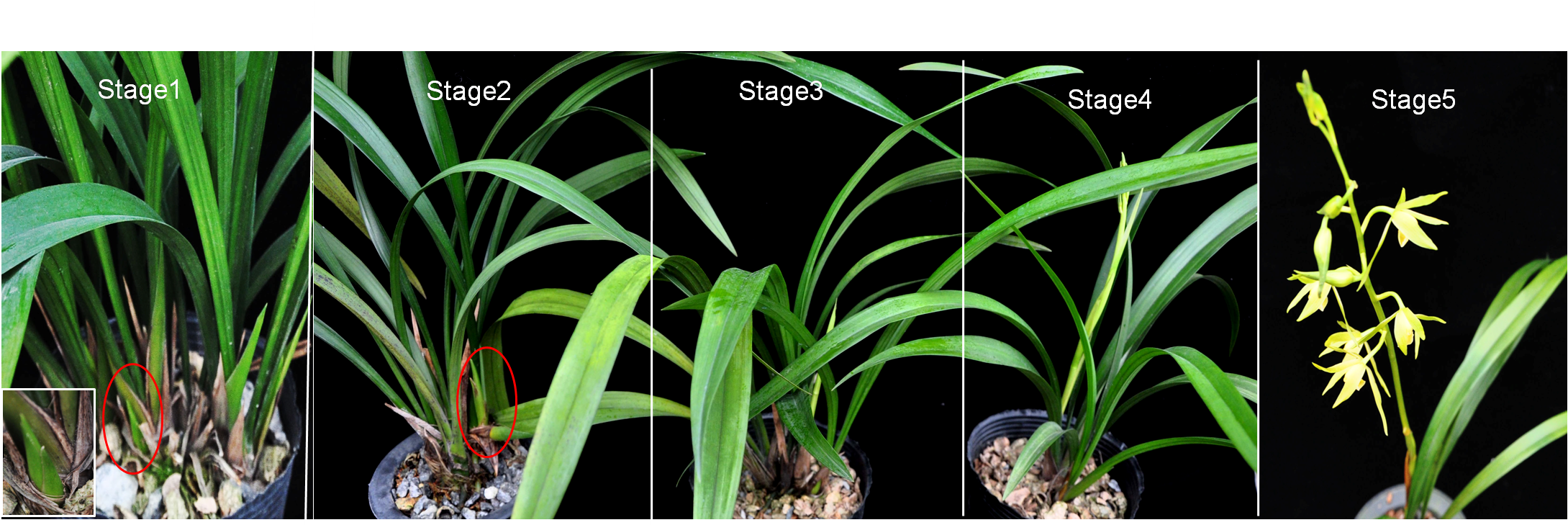

Supplement: Supplementary file 5 — Figure S1. Floral development stages of Cymbidium sinense. (TIF 3245 kb) [file 12864_2019_5425_MOESM5_ESM.tif]

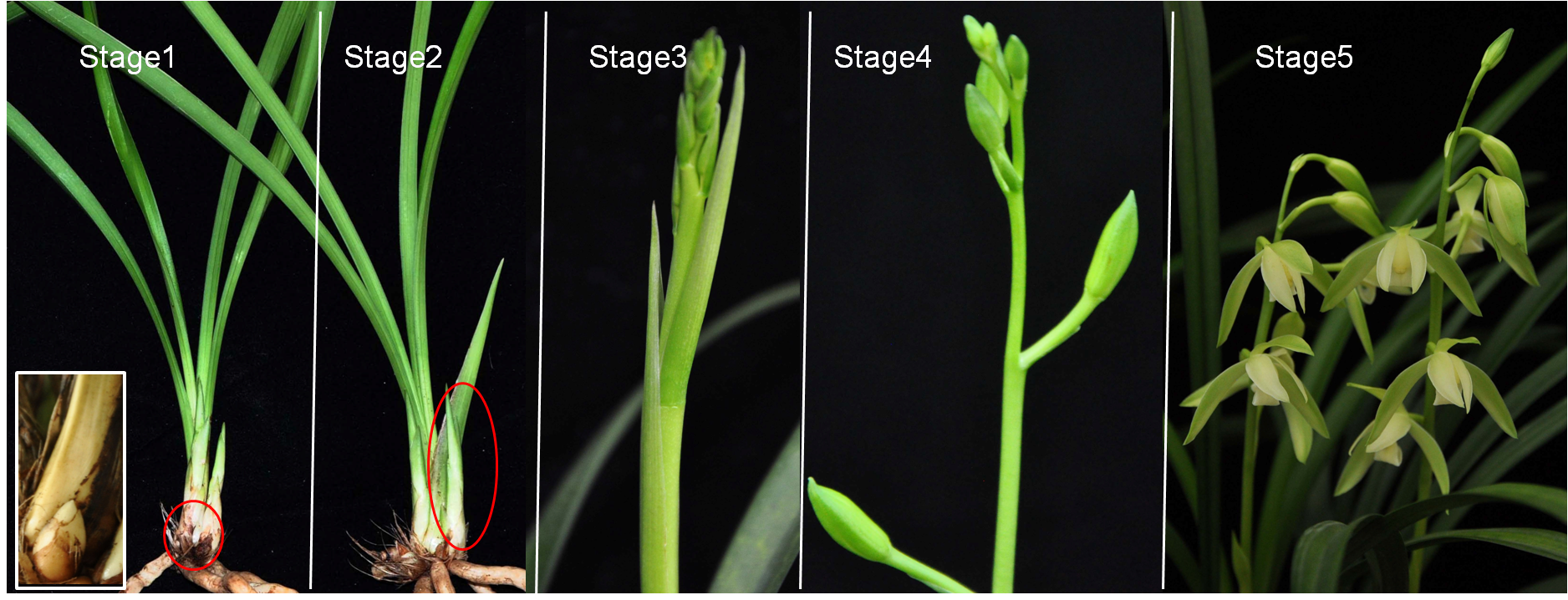

Supplement: Supplementary file 6 — Figure S2. Floral development stages of Cymbidium ensifolium. (TIF 3232 kb) [file 12864_2019_5425_MOESM6_ESM.tif]

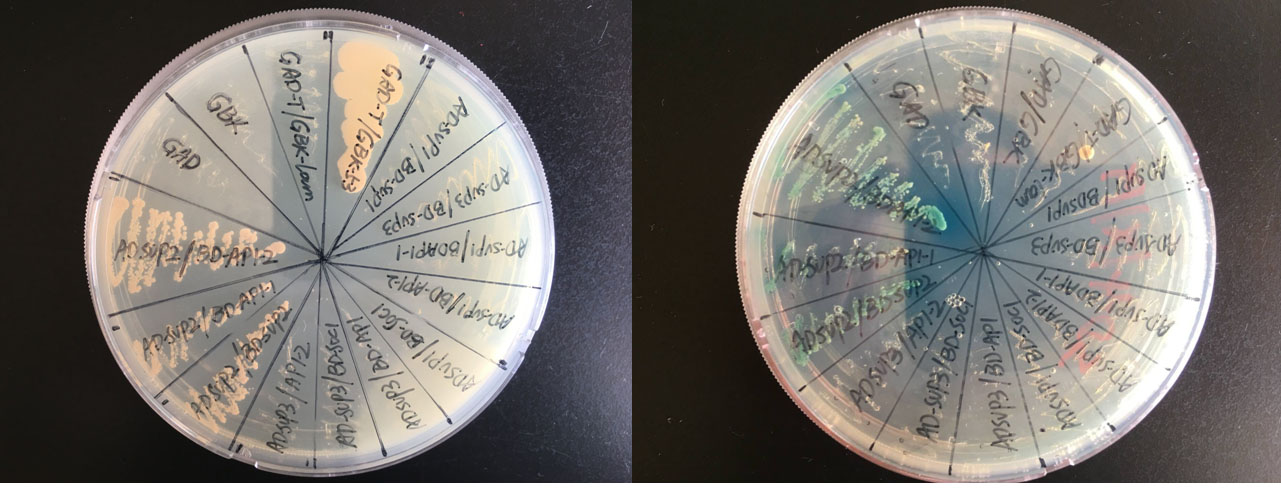

Supplement: Supplementary file 7 — Figure S3. Homodimerization and Heterodimerization of Cymbidium goeringii MADS-box proteins. (JPG 142 kb) [file 12864_2019_5425_MOESM7_ESM.jpg]
